# Investigating the impact of botulinum toxin type a on the migration of normal human dermal fibroblasts: An in vitro wound healing assay

**DOI:** 10.1111/jocd.16406

**Published:** 2024-06-19

**Authors:** Wilai Thanasarnaksorn, Cherrin Pomsoong, Phongthon Kanjanasirirat, Kedchin Jearawuttanakul, Suparerk Borwornpinyo, Suradej Hongeng, Yanisa Ratanapokasatit, Teerapong Rattananukrom

**Affiliations:** ^1^ Division of Dermatology, Department of Internal Medicine, Faculty of Medicine Ramathibodi Hospital, Mahidol University Bangkok Thailand; ^2^ Division of Dermatology, Chulaporn International College of Medicine Thammasat University Pathum Thani Thailand; ^3^ Excellence Center for Drug Discovery, Faculty of Science Mahidol University Bangkok Thailand; ^4^ Department of Biotechnology, Faculty of Science Mahidol University Bangkok Thailand; ^5^ Department of Pediatrics, Faculty of Medicine, Ramathibodi Hospital Mahidol University Bangkok Thailand

**Keywords:** botulinum toxin type a, fibroblast migration, in vitro assay, scar prevention, wound healing

## Abstract

**Background:**

Botulinum toxin A (BoNT‐A) is widely utilized in the management of hypertrophic and keloid scars. One proposed mechanism for scar prevention involves the inhibition of fibroblast migration in scars by BoNT‐A. However, the data regarding the effect of BoNT‐A on the migration of normal human dermal fibroblasts (NHDF) is limited.

**Objectives:**

The aim of this study was to investigate the inhibitory effect of different types and dilutions of BoNT‐A on the migration of NHDF.

**Methods:**

In vitro scratch wound assay, NHDF cells were cultured, incubated, and subjected to scratching using a sterile tip. Subsequently, the scratched NHDF monolayer was treated with different types of BoNT‐A, including onabotulinumtoxinA (ONA), incobotulinumtoxinA (INCO), prabotulinumtoxinA (PRABO), or letibotulinumtoxinA (LETI), at varying concentrations of 10, 20, 25, 40, 50, and 100 units/milliliter (U/mL). Additionally, abobotulinumtoxinA (ABO) was administered at concentrations of 33, 50, 66, 71, 100, 150, 300, and 500 U/mL. Normal saline solution (NSS) served as a negative control. The extent of NHDF migration was evaluated by comparing each dilution of BoNT‐A with the controls using high‐content imaging at the 48‐h time point. Furthermore, the viability of the of NHDF was assessed.

**Results:**

The concentrations of 25, 40, and 50 U/mL of ONA (*p* < 0.001) and 25 U/mL of LETI (*p* < 0.05) demonstrated significantly inhibited NHDF migration in comparison to the control group. Conversely, all dilutions of PRABO, INCO, and ABO exhibited comparable NHDF migration to that of the control group. Regarding NHDF viability, no significant decrease was observed across any of the BoNT‐A types and dilutions.

**Conclusion:**

Different types and dilutions of BoNT‐A demonstrated variable inhibitory effects on NHDF migration in vitro. The selection of BoNT‐A formulation may significantly impact the clinical outcome of scar prevention related to fibroblast migration.

## 1 INTRODUCTION

Botulinum toxin A (BoNT‐A) is a neurotoxin derived from Clostridium botulinum bacteria. Its mechanism of action involves chemodenervation by targeting presynaptic neurons and blocking the release of acetylcholine at the neuromuscular junction. As a result, muscle relaxation occurs, leading to therapeutic effects.^1^, ^2^ BoNT‐A has received clinical approval for the treatment of several medical conditions, including cervical dystonia, blepharospasm, axillary hyperhidrosis, chronic migraine, strabismus, and frown lines.^3^, ^4^

In recent years, there has been growing utilization of BoNT‐A for the prevention and treatment of hypertrophic and keloid scars.^5^, ^6^ BoNT‐A has been reported to exhibit inhibitory effects on the growth of pathologic scar tissue, leading to an improvement in their appearance when used in clinical practice. In addition to its chemoimmobilization properties, BoNT‐A is proposed to reduce scar formation by modulating various activities of fibroblasts. This includes influencing their migration, proliferation, and fibrotic pathways. BoNT‐A can also impact the differentiation of fibroblasts into myofibroblasts during the process of wound healing. Moreover, BoNT‐A has the ability to suppress the expression of transforming growth factor beta (TGF‐β) in fibroblasts, a key regulator of their migration and proliferation. These multifaceted effects of BoNT‐A on fibroblast behavior contribute to its potential in scar prevention and management.^7^, ^8^

Wound healing is a complex and sequential process that involves several distinct phases, including coagulation, inflammation, proliferation, and remodeling. Each phase plays a critical role in the repair and regeneration of damaged tissue.^9^ However, the exact pathogenesis of pathologic scars, such as hypertrophic and keloid scars, is not yet fully understood. While aberrations at various stages of wound healing, particularly in the migratory and proliferative phases of fibroblasts, are thought to contribute to the excessive production of extracellular matrix and the formation of these abnormal scars, the underlying mechanisms and factors involved require further investigation.^10^, ^11^

Previous in vitro studies have provided evidence of the inhibitory effects of BoNT‐A on the migration of fibroblast cells derived from human hypertrophic and keloid scars.^8^, ^12^ It has been hypothesized that the reduction in fibroblast migration towards the injured area is associated with a decrease in the formation of pathologic scars. Consequently, the inhibition of fibroblast migration represents one of the potential mechanisms by which BoNT‐A may contribute to scar prevention. However, despite these hypotheses, there is still limited available data regarding the specific effect of BoNT‐A on normal human dermal fibroblasts (NHDF). Therefore, the objective of our study is to investigate the inhibitory effect of different types and dilutions of BoNT‐A on NHDF using an in vitro scratch wound assay.

## 2 MATERIALS AND METHODS

### 2.1 Cellular wound healing assays and cytotoxicity assays

Cellular wound healing assays and cytotoxicity assays were performed to evaluate the effects of the BoNT‐A preparations. The cellular wound healing assay measured the migration of cells in a scratch wound model, while the cytotoxicity assay assessed the potential toxicity of the BoNT‐A preparations on the cells.

NHDF obtained from PromoCell, Heidelberg, Germany, were selected at a density of 20 000 cells per well in a 96‐well clear flat‐bottom plate. The cells were cultured in Fibroblast Growth Medium 2 (PromoCell, Heidelberg, Germany) and incubated in a humidified carbon dioxide (CO_2_) incubator at 37°C with 5% CO2 for 24 h. Subsequently, the NHDF cells were scratched using a sterilized 200‐microliter pipette tip to create a monolayer wound. The cells were then washed once with phosphate‐buffered saline (PBS). Varying concentrations of five different brands of BoNT‐A were administered to the cells. After 48 h, the cells were captured and analyzed using a high‐content imaging system (Operetta®, PerkinElmer, Waltham, MA, USA). The 48‐h time point was chosen as it allows fibroblasts to naturally migrate and close the wound gap.

Additionally, cytotoxicity was assessed using a 3‐(4,5‐dimethylthiazol‐2‐yl)‐2,5‐diphenyltetrazolium bromide (MTT) assay. In this assay, the MTT reagent, a yellow tetrazolium salt, is reduced to purple formazan crystals by mitochondrial reductase in live cells. The insoluble formazan crystals are then dissolved using a solubilization solution, and the resulting colored solution is quantified by measuring the absorbance at 570 nanometers using a MultiMode Microplate Reader (ENVISION®, PerkinElmer, Waltham, MA, USA). A higher absorbance indicates a greater number of viable cells. The MTT assay was employed to measure cellular metabolic activity as an indicator of cell viability, proliferation, and cytotoxicity for each concentration of the different BoNT‐A preparations.

### 2.2 The preparation and testing of BoNT‐A

Normal saline solution (NSS) was employed as a negative control in this study. The monolayer of scratched NHDF cells in each well plate was treated with five different solutions of BoNT‐A: onabotulinumtoxinA (ONA; Botox®, Allergan Inc.), abobotulinumtoxinA (ABO; Dysport®, Ipsen Biopharm Ltd.), incobotulinumtoxinA (INCO; Xeomin®, Merz Pharmaceuticals, GmbH), prabotulinumtoxinA (PRABO; Nabota®, Daewoong Pharmaceutical), and letibotulinumtoxinA (LETI; Hugel, Hugel Inc.). These BoNT‐A solutions were administered at various dilutions commonly used by physicians in clinical practice, as outlined in Table 1.

**TABLE 1:** Different types and dilutions of botulinum toxin A in this study.

Botulinum toxin A	Dilution	Normal saline solution (mL)	Units/mL
100 unit vial of OnabotulinumtoxinA (ONA) IncobotulinumtoxinA (INCO) PrabotulinumtoxinA (PRABO) LetibotulinumtoxinA (LETI)	1:1	1	100
	1:2	2	50
	1:2.5	2.5	40
	1:4	4	25
	1:5	5	20
	1:10	10	10
500 unit vial of AbobotulinumtoxinA (ABO)	1:1	1	500
	1:1.7	1.7	300
	1:3.3	3.3	150
	1:5	5	100
	1:7	7	71
	1:7.5	7.5	67
	1:10	10	50
	1:15	15	33

Photographic images of NHDF migration were captured at different time points: baseline, immediate, 2 h, 4 h, 12 h, 24 h, and 48 h after the administration of the BoNT‐A solutions. The migrated area of NHDF was assessed and expressed as a percentage of the initially scratched area at baseline, as fibroblasts in the absence of BoNT‐A administration generally close the gap within 48 h. A lower percentage of migration indicates a higher inhibitory effect of BoNT‐A. The migrated area of NHDF treated with each BoNT‐A preparation was then compared to the control group. Each dilution of the BoNT‐A preparations was tested in triplicate, resulting in three samples for each dilution.

### 2.3 Statistical analysis

All data analysis was performed using GraphPad Prism Version 9 (Domatics, Boston, MA, USA). Numerical data were presented as mean ± standard deviation. The statistical differences between two groups were assessed using the t‐test, while ordinary one‐way analysis of variance (ANOVA) was utilized for comparisons among multiple groups. A significance level of *p* < 0.05 was considered statistically significant.

## 3 RESULTS

### 3.1 The migration of fibroblasts following the administration of BoNT‐A

At the 48‐h time point, the control group exhibited a fibroblast migrated area of 100%. However, NHDF cells treated with ONA and LETI showed significantly reduced fibroblast migration compared to the controls. Specifically, the dilutions of 25, 40, and 50 units/milliliter (U/mL) of ONA exhibited significant inhibition of fibroblast migration (*p* < 0.001), with migration areas at 48 h measured at 59.00 ± 6.92%, 66.17 ± 2.53%, and 56.73 ± 6.23%, respectively. Similarly, the concentration of 25 U/mL of LETI also demonstrated reduced fibroblast migration compared to the controls (*p* < 0.05), with a migration area of 66.63 ± 4.35%. On the other hand, PRABO, INCO, and ABO did not show significant differences in fibroblast migrated area compared to the controls (Figure 1). The accompanying images illustrate the scratched area at baseline and the fibroblast migrated area at 48 h under the influence of each BoNT‐A preparation.

**FIGURE 1:**
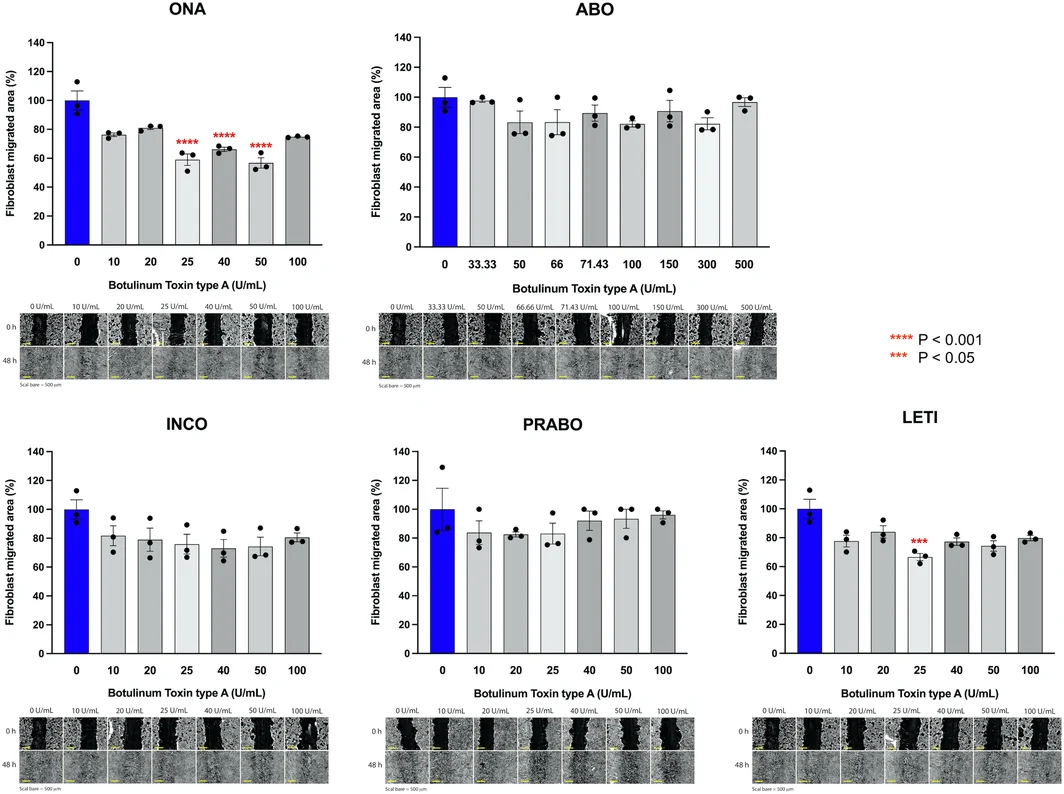
The fibroblast migrated area at 48 h with different dilutions of botulinum toxin A (BoNT‐A); The x‐axis represents the varying concentrations of BoNT‐A in units/milliliter (U/mL), while the y‐axis represents the percentage of fibroblast migrated area. The bar charts show the scratched area at baseline and the fibroblast migrated area at 48 h under the influence of each BoNT‐A preparation. Significant differences are denoted by asterisks: ***p < 0.05, ****p < 0.001. The abbreviations used in the figure are as follows: ABO (abobotulinumtoxinA), INCO (incobotulinumtoxinA), LETI (letibotulinumtoxinA), ONA (onabotulinumtoxinA), and PRABO (prabotulinumtoxinA).

### 3.2 Cytotoxicity analysis

To confirm the potential cytotoxicity of BoNT‐A on NHDF, the number of viable cells was assessed at the 48‐h time point following treatment with BoNT‐A using the MTT assay. The results demonstrated that there was no significant decrease in the number of NHDF across all types and dilutions of BoNT‐A tested (Figure 2). These findings indicate that none of the BoNT‐A preparations exhibited cytotoxic effects on NHDF, affirming their safety profile in this regard.

**FIGURE 2:**
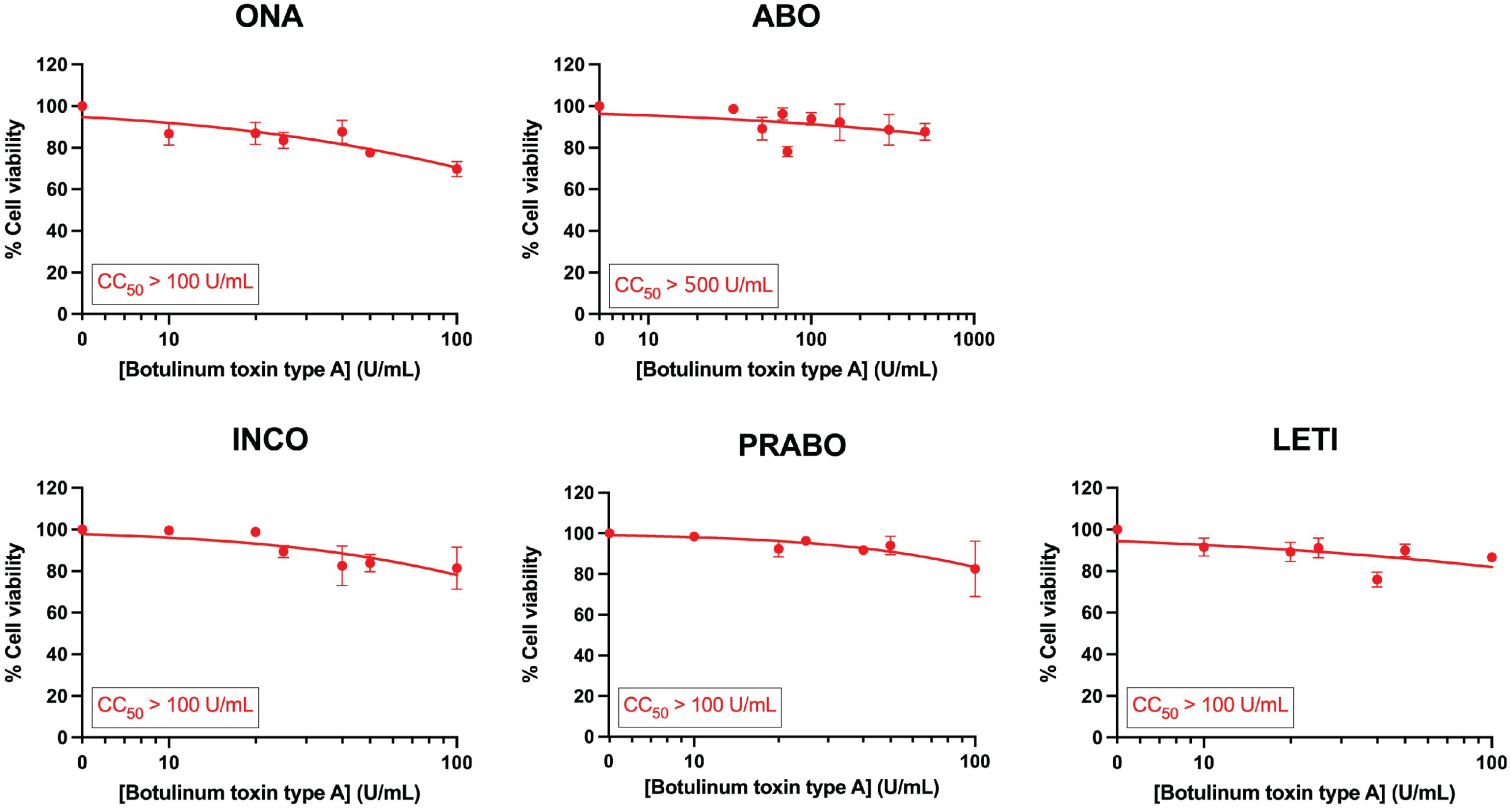
The results of the cell viability assay at 48 h with different dilutions of botulinum toxin A (BoNT‐A); The x‐axis represents the varying concentrations of BoNT‐A in units/milliliter (U/mL), while the y‐axis represents the percentage of fibroblast viability. The abbreviations used in the figure are as follows: ABO (abobotulinumtoxinA), CC50 (50% cytotoxicity concentration), INCO (incobotulinumtoxinA), LETI (letibotulinumtoxinA), ONA (onabotulinumtoxinA), and PRABO (prabotulinumtoxinA).

## 4 DISCUSSION

The proposed mechanism of action for BoNT‐A in scar prevention involves its ability to block neuromuscular transmission and induce chemoimmobilization, resulting in reduced muscle tension and inflammation during the wound healing process.^8^ In addition to its effects on neuronal cells, BoNT‐A has been demonstrated to impact various nonneuronal cell types, including dermal fibroblasts, epidermal keratinocytes, epithelial cells, and endothelial cells. BoNT‐A can elicit specific biological effects on these cell types, leading to phenotypic changes.^13^ Regarding fibroblasts, BoNT‐A has shown inhibitory effects on several aspects of their function. It can inhibit fibroblast migration and proliferation, thus preventing excessive cell division; reducing the overall number of fibroblasts within the wound area; limiting their movement into the wound bed; and potentially restricting the extent of scar formation. Additionally, BoNT‐A can influence the migration and differentiation of fibroblasts into myofibroblasts, which are contractile cells responsible for wound contraction and scar development. By inhibiting fibroblast migration and differentiation, BoNT‐A can help minimize the contractile forces exerted during wound healing, leading to a reduction in scar formation.^7^, ^8^, ^14^ Furthermore, studies have indicated that BoNT‐A alters the cell cycle distribution of fibroblasts by decreasing the number of cells in the G2‐M phase, resulting in reduced fibroblast migration and proliferation.^15^, ^16^ Moreover, BoNT‐A can modulate collagen activity, which is produced by fibroblasts and is the primary component of scar tissue. It regulates collagen synthesis and degradation, leading to a reduction in scar hyperplasia and promoting a more balanced and controlled collagen remodeling process during wound healing.

Several studies have investigated the effects of BoNT‐A on proliferation in hypertrophic scars and keloids, and the majority of these studies have shown that BoNT‐A reduces proliferation in hypertrophic dermal fibroblasts (HDF) and keloid dermal fibroblasts (KDF) in in vitro models.^7^ Furthermore, in vivo studies have demonstrated a decrease in collagen deposition and fibril thickness in animal models of hypertrophic scars, such as rats and rabbits, following treatment with BoNT‐A.^14^, ^17^, ^18^

As we mention, the mechanism of scars is related to fibroblast migration. The key factors would be the inhibition of fibroblast migration, including HDF, KDF, and NHDF, leading to a decrease in scar formation in the proliferation and remodeling phases of the wound healing process. Two previous studies have demonstrated that BoNT‐A suppresses the migration of HDF and KDF using the scratch wound assay.^8^, ^12^ However, the available data regarding the effect of different types and dilutions of BoNT‐A on NHDF migration is still limited. Our study investigated the potential inhibitory effects of different BoNT‐A preparations on NHDF migration using an in vitro scratch wound assay.

To the best of our knowledge, this is the in vitro study to investigate the inhibitory effect of different types and dilutions of BoNT‐A on the migration of NHDF, as well as their impact on cell viability. The results of this study revealed that concentrations of 25, 40, and 50 U/mL of ONA, as well as 25 U/mL of LETI, significantly inhibited fibroblast migration compared to other concentrations. In terms of safety evaluation, our study observed no cytotoxic effects of BoNT‐A on dermal fibroblasts, which is consistent with previous findings in the literature.^19^, ^20^, ^21^ These findings contribute to our understanding of the potential therapeutic effects of BoNT‐A on fibroblast migration and emphasize the safety of these treatments in relation to cell viability.

Numerous studies have provided evidence supporting the efficacy of BoNT‐A in preventing hypertrophic scars and keloids. Furthermore, several studies have demonstrated the positive impact of BoNT‐A on improving the appearance of scars in postoperative surgical wounds.^5^ However, it is important to note that the concentration of BoNT‐A used in these studies has varied. For instance, ONA has been administered at concentrations ranging from 10 to 100 U/mL, while lanbotulinumtoxinA, which is not available in Thailand, has been utilized at concentrations of 10–50 U/mL. Other BoNT‐A preparations are also used in various dilutions.^5^, ^6^, ^22^ Considering the findings of our study, it is suggested that concentrations of 25, 40, and 50 U/mL of ONA, as well as 25 U/mL of LETI, may be the optimal choices for scar prevention in clinical practice.

One limitation of our study is that we used a single cell line of normal human fibroblasts. To obtain a more comprehensive understanding of the effects of BoNT‐A, future studies should investigate its effects on fibroblasts derived from hypertrophic scars or keloid scars, using different types and dilutions of BoNT‐A. By utilizing these specific cell lines, we can gain valuable insights into the potential therapeutic benefits of BoNT‐A in the context of pathological scar formation. This would contribute to a more robust assessment of BoNT‐A efficacy and help guide its clinical application in scar management.

## 5 CONCLUSION

This study represents the investigation of the effects of different preparations of BoNT‐A on NHDF migration. Our findings demonstrate that various types and dilutions of BoNT‐A exert variable inhibitory effects on fibroblast migration in vitro. These results highlight the importance of carefully considering the selection of BoNT‐A in clinical practice, as it may significantly impact the clinical outcomes of scar prevention strategies. Further research is warranted to elucidate the underlying mechanisms and establish standardized guidelines for the optimal use of BoNT‐A in scar management.

## AUTHOR CONTRIBUTIONS

T.R., W.T., C.P., P.K., S.B., S.H., and Y.R. participated in the conceptualization and performed the research. T.R., W.T., S.B., and S.H. designed the research study. P.K., K.J., and S.B. contributed essential reagents or tools. Y.R., P.K., and K.J. analyzed the data. Y.R and T.R. wrote the paper. All authors have read and approved the final manuscript.

## CONFLICT OF INTEREST STATEMENT

All authors declare no conflict of interest for this project.

## ETHICS STATEMENT

In vitro studies do not require formal ethics approval, and neither do the policies and regulations of the institution necessitate such approval. No studies on animals or humans.

## Data Availability

Data and study protocol are available from the corresponding author upon reasonable requests.
